# The Relentless Recurrence of Diffuse Alveolar Hemorrhage in Catastrophic Antiphospholipid Syndrome and Lupus: A Therapeutic Challenge

**DOI:** 10.7759/cureus.62635

**Published:** 2024-06-18

**Authors:** Aruni Rahman, Mashal Nathani, Michael Malekan

**Affiliations:** 1 Internal Medicine, New York-Presbyterian Brooklyn Methodist Hospital, Brooklyn, USA; 2 Internal Medicine, Medical University of the Americas, Charlestown, KNA; 3 Rheumatology, New York-Presbyterian Brooklyn Methodist Hospital, Brooklyn, USA

**Keywords:** bleeding, pulmonary hemorrhage, alveolar hemorrhage, systemic lupus erythematosus antiphospholipid antibodies, catastrophic antiphospholipid syndrome

## Abstract

Diffuse alveolar hemorrhage (DAH), a rare complication of coexisting antiphospholipid syndrome (APS) and systemic lupus erythematosus (SLE), poses significant diagnostic and therapeutic challenges, especially with recurrent episodes. We present a 27-year-old male with catastrophic APS and SLE who experienced acute respiratory failure and hemoptysis due to DAH. Despite aggressive therapy with immunosuppressants, plasma exchange, and anticoagulation, he had recurrent DAH episodes requiring repeated admissions. Early recognition, multidisciplinary management, and utilization of effective targeted therapies, such as intravenous immunoglobulin, in refractory cases are crucial for improving outcomes in this challenging complication.

## Introduction

Objective

By presenting a case of a patient with known catastrophic antiphospholipid syndrome (CAPS) and systemic lupus erythematosus (SLE) who developed recurring and severe diffuse alveolar hemorrhage (DAH), the aim is to raise awareness of this atypical complication among clinicians and the utilization of intravenous immunoglobulin (IVIG) to successfully treat and prevent recurrence in refractory cases.

Background

DAH is a rare but potentially life-threatening complication of autoimmune disorders [1]. When faced with the coexistence of SLE and CAPS, a potentially fatal variant of antiphospholipid syndrome (APS), diagnostic and therapeutic challenges arise, particularly when DAH recurs despite appropriate management. With CAPS, patients experience simultaneous thromboses rapidly, typically affecting small vessels in several vital organs [2,3]. Treatment for DAH involves multidisciplinary management, treatment of underlying disease, and effective, rapid local hemostasis [4]. Early recognition and prompt initiation of targeted therapies are crucial for improving outcomes in this challenging complication.

## Case presentation

**Figure 1:**
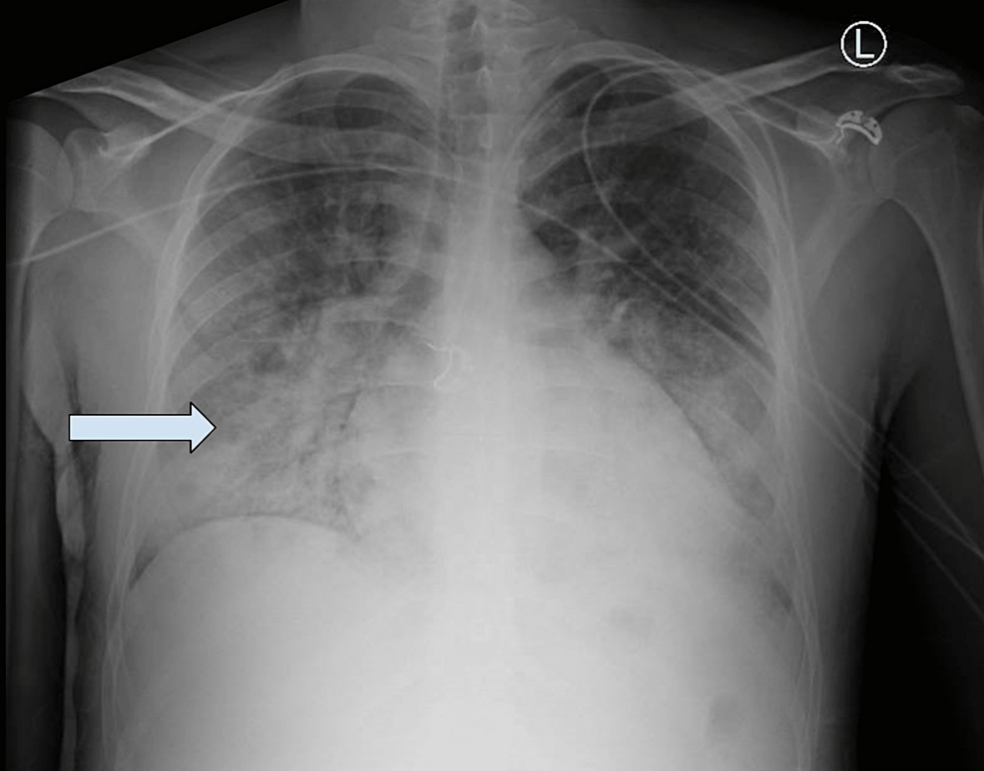
Chest XR (readmission CXR): New subtle patchy area of airspace opacity in the right mid lung with significant interval improvement in patchy airspace opacities in the bilateral lower lobes.

The patient is a 27-year-old male with known SLE, chronic kidney disease, and CAPS complicated by cardiac arrest with massive pulmonary hemorrhage (treated with rituximab, discontinued due to severe hypersensitivity reaction) presenting with myalgias, arthralgias, and macular rash for two days. On admission, the patient developed hemoptysis. Chest X-ray (CXR) (Figure 1) showed diffuse opacities bilaterally, and he was upgraded to the intensive care unit (ICU) for the treatment of suspected DAH. The patient received five sessions of plasmapheresis, two sessions of hemodialysis, pulse-dose steroids, and four doses of mycophenolate mofetil. He was started on a steroid taper, transitioned to cyclophosphamide, and discharged home.

**Figure 2:**
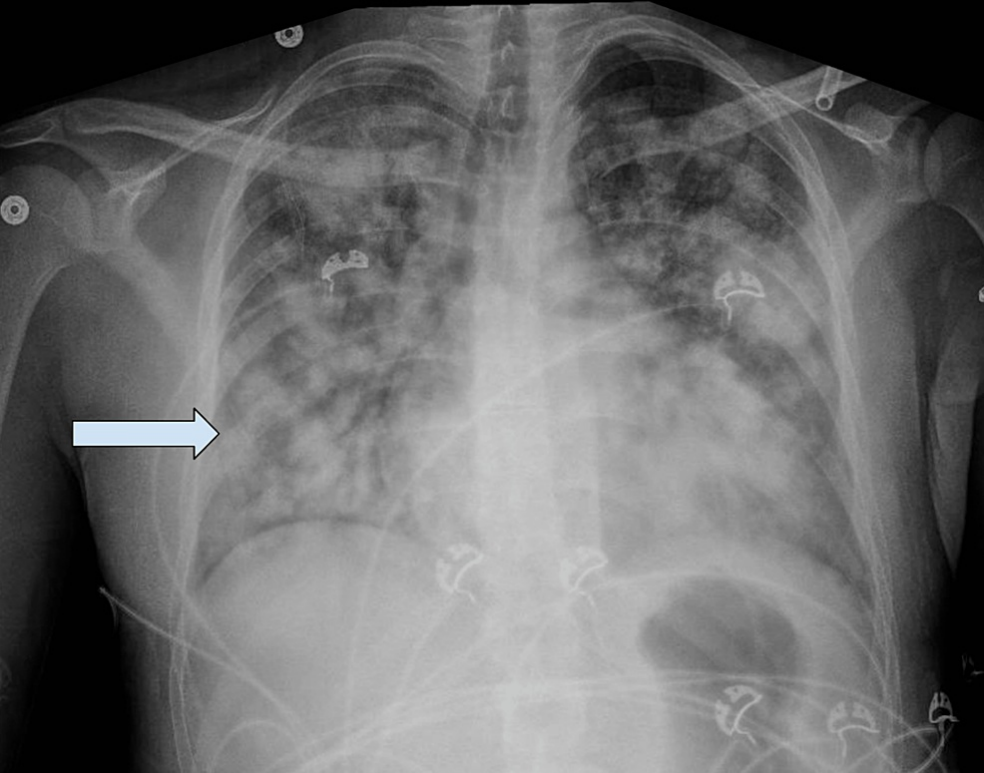
Chest XR: Diffuse multiple fluffy nodular densities are seen with the largest one seen in the right upper lobe and periphery of the left mid lung which has the appearance of multiple fluffy nodular infiltrates with superimposed pulmonary edema.

The following day, the patient returned with recurrent hemoptysis. CXR (Figure 2) obtained showed diffuse densities with superimposed pulmonary edema. The patient was intubated and restarted on pulse-dose steroids. The patient received hemodialysis, empiric broad-spectrum antibiotics, and seven sessions of plasmapheresis and was eventually extubated. Shortly after, hemoptysis reoccurred. He received plasmapheresis with the initiation of IVIG with improvement of symptoms. He transitioned to oral steroids, had complete resolution of his hemoptysis, and was discharged to acute rehabilitation.

## Discussion

The coexistence of CAPS and SLE presents a unique set of challenges, particularly when complicated by DAH. CAPS affects about 1% of patients with APS and has been suggested to have a 30% mortality rate, despite appropriate treatment [5]. Considering this, there is very little data regarding the incidence of DAH in the setting of CAPS. This potentially life-threatening complication highlights the complex interplay between these two autoimmune disorders and their potential impact on the pulmonary system [6]. 

The most common proposed mechanism of DAH in the setting of APS is antibody-associated pulmonary capillaritis [5,7]. Additionally, in situ microvascular thrombi may be a mechanism behind DAH [5]. The presence of antiphospholipid antibodies is believed to promote a procoagulant state, leading to the formation of microthrombi within the pulmonary vasculature. The inflammatory processes associated with SLE may contribute to endothelial injury and increased vascular permeability. Furthermore, immune complex deposition and complement activation can play a role in the pathogenesis of DAH [8].

The treatment of recurrent DAH in patients with coexisting CAPS and SLE targets the underlying autoimmune processes and the alveolar hemorrhage itself. In patients with a refractory disease course, IVIG and plasma exchange should be considered [4]. Plasma exchange has been employed to remove pathogenic autoantibodies and immune complexes from circulation, potentially halting the progression of alveolar hemorrhage. Although the role of anticoagulation in the setting of DAH remains controversial, in these patients, anticoagulation is often considered to prevent the formation of new microthrombi and limit further alveolar-capillary membrane disruption. Many CAPS patients receive triple therapy which includes anticoagulation, corticosteroids, and plasma exchange ± IVIG [9,10]. In this patient, IVIG was successfully administered and resulted in overall improvement and a decrease in rehospitalizations. 

In refractory cases, immunological therapies must be considered [4]. Rituximab, a monoclonal antibody targeting B cells, may be an option in patients with DAH with autoimmune conditions [4]. Additionally, the introduction of eculizumab, a C5 complement inhibitor, may prove effective in achieving disease control and reducing the frequency of DAH episodes [4]. In this case, the patient experienced allergic reactions to rituximab and was unable to tolerate the medication. This may be an additional setting in which IVIG may be preferable over other therapies. This case serves as a poignant reminder of the challenges posed by CAPS and its associated complications.

## Conclusions

Recurrent DAH in the setting of CAPS and SLE represents a formidable diagnostic and therapeutic challenge. Despite the resolution of DAH, the patient's complex medical history and organ involvement underscore the need for close outpatient monitoring and continued management of his underlying conditions. Early recognition, multidisciplinary management, and successful use of immunological therapies, such as IVIG, are crucial in navigating the challenges posed by this rare, but devastating complication.
